# Perinatal Depression and Patterns of Attachment: A Critical Risk Factor?

**DOI:** 10.1155/2015/105012

**Published:** 2015-12-20

**Authors:** Valentina Meuti, Franca Aceti, Nicoletta Giacchetti, Giuseppe Mattia Carluccio, Michela Zaccagni, Isabella Marini, Orazio Giancola, Paola Ciolli, Massimo Biondi

**Affiliations:** ^1^Department of Neurology and Psychiatry, Policlinico Umberto I, Sapienza University of Rome, Viale dell'Università 30, 00185 Rome, Italy; ^2^Department of Social Science, Faculty of Political Science, Sociology, Communication, Sapienza University of Rome, Via Salaria 113, 00198 Rome, Italy; ^3^Department of Gynecological, Obstetric and Urological Science, Policlinico Umberto I, Sapienza University of Rome, Viale del Policlinico 155, 00161 Rome, Italy

## Abstract

*Background*. This study aims to verify if the presence and severity of perinatal depression are related to any particular pattern of attachment.* Methods*. The study started with a screening of a sample of 453 women in their third trimester of pregnancy, who were administered a survey data form, the Edinburgh Postnatal Depression Scale (EPDS) and the Experience in Close Relationship (ECR). A clinical group of subjects with perinatal depression (PND, 89 subjects) was selected and compared with a control group (C), regarding psychopathological variables and attachment patterns.* Results*. The ECR showed a prevalence of “Fearful-Avoidant” attachment style in PND group (29.2% versus 1.1%, *p* < 0.001); additionally, the EPDS average score increases with the increasing of ECR dimensions (Avoidance and Anxiety).* Conclusion*. The severity of depression increases proportionally to attachment disorganization; therefore, we consider attachment as both an important risk factor as well as a focus for early psychotherapeutic intervention.

## 1. Introduction

Perinatal depression (PND) manifests in a number of different ways, varying in severity and period of onset: prenatal depression, “baby blues,” and postpartum depression. It has a prevalence of 10–20% [1] and can occur during pregnancy, especially in the third trimester, or from several weeks to several months after childbirth [2]. Depressive symptoms experienced in perinatal period are similar to classic symptoms of depression, including depressed mood, loss of interest or enjoyment, and reduced energy [3]. Even if depressive features may show a spontaneous remission, many women are still depressed one year after childbirth; effective pharmacological and nonpharmacological treatments are available, but both patients and their families often neglect depressive features during the perinatal period.

In order to plan more effective strategies to quickly identify and intervene in perinatal depression, it is necessary to consider the underlying pathogenetic pathways of the disorder. The literature has not identified a specific cause of perinatal depression. In fact, the actual framework of risk factors points to a multifactor model in which it is necessary to take into account the interaction between the biological, psychological, and social aspects [4–12].

The risk factors present in the literature are classified into three categories, according to their effect size: strong-moderate, moderate, and weak. The strongest predictors of perinatal depression are represented by depression or anxiety during pregnancy or a history of mental health problems (particularly depression) [13, 14]. Life stressors and lack of social support are factors that are regarded as strong-moderate; psychosocial and marital problems have a moderate effect size, while obstetric and socioeconomic factors have a low effect size [14, 15].

Moreover, in the latest years the assessment of the quality of attachment has assumed a particular importance in the study of psychological risk factors that predispose the development of affective disorders [16].

Attachment theory assumes that people have an innate need for social support and interpersonal connection [17]. Based on the quality of one's early interpersonal experiences, such as the responsiveness of caregivers, the child formulates internal working models, which include expectations of whether support will be available and how to most effectively obtain the needed support [18–20]. Though heavily influenced by caregiving provided during infancy, attachment orientation is continually shaped throughout life [21].

Those internal working models continue and heavily influence adult behaviors in interpersonal relationships, so that the attachment in childhood can address the ways in which the subject will establish a relationship and build emotional bonds with partners in adulthood (romantic attachment). Thus, individuals who grow up with supportive and responsive parents develop* secure* attachments and positive working models of relationships; in the romantic relationship, they will expect that support is available when needed and that directly asking for support is likely to result in its provision.

In contrast,* insecure* attachments are characterized by the belief that support will not be available or will be inconsistently available as well as ineffective need-fulfilling behaviors. In the romantic relationship, insecure attachment orientations are often described as involving high levels of* Anxiety* and/or* Avoidance* [22]; high levels of discomfort and mistrust of others as reliable attachment figures drive both. Individuals with anxious attachments highly stress, and even overvalue, the importance of torque relationships and engage in maladaptive behaviors to ensure that those relationships are maintained. Those with avoidant attachments devalue and distance themselves from relationships with a partner to minimize their interpersonal discomforts [23–25]. Consequently, avoidant individuals may identify sources for their gratification needs in work or academic achievements [26], rather than interpersonal relationships [23, 25, 27]. Thus, individuals with anxious and avoidant attachments may be vulnerable to social stressors in a different way than achievement-related stressors and are more likely to experience problematic outcomes because they are less likely to obtain the needed support [28].

Because of this, the insecure attachment is considered a persistent point of vulnerability for social maladjustment or dysfunction and even for psychopathology. Dozier et al. demonstrate that an insecure attachment increases as many as four times the possibility of developing any mental disorder, in particular a mood disorder [29]. Nevertheless, little attention has been paid to the area of perinatal maternal distress: this is surprising, given the close association between the organization of internal working models and biological, affective, and identity aspects characterizing pregnancy and motherhood.

The transition to parenthood can be considered a stressful life event that activates the attachment system. Pregnancy, in particular in its third trimester, is a highly vulnerable period in which there are many transformations from physical, psychological, and relational points of view, which implicate a considerable psychological adaptation and reorganization of interpersonal relationships, including those with partner: during this transition, some women can develop affective disorders of varying intensity [30]. Data suggests the idea that women with an insecure attachment pattern have a higher risk of suffering from an affective disorder after childbirth as a result of the activation of the internal working models related to negative representations of self and others [31–40].

In light of this theoretical framework, this study is the first phase of a longitudinal research aimed at identifying predictors of perinatal depression. In this phase, we focus on a particular kind of psychological risk factors—the quality of attachment—to verify its relevance among the severity of illness. In particular, the specific goal of this paper is to verify if the presence and the severity of PND are related to any particular pattern of attachment.

## 2. Materials and Methods

### 2.1. Study Population

The study started by conducting a screening, performed by the “Perinatal Disorder Unit” affiliated with the Department of Psychiatry of Policlinico Umberto I in Rome, of pregnant outpatients and inpatients admitted by the Department of Gynaecology and Obstetrics of the same hospital between March 2009 and June 2012 [41]. A sample of 453 consecutive subjects were selected, including pregnant women between the ages of 18 and 45 years in their third trimester of pregnancy. The exclusion criteria were as follows: the refusal to provide informed consent, being under the age of 18 years, the presence of a diagnosis of mental retardation or schizophrenia, poor knowledge of Italian, or other verbal communication limitations compromising the ability of the subject to follow the research protocol. Before being enrolled in the study, participants were informed of the nature and objectives of the research. Enrollment was voluntary and both verbal and written consents were obtained. The study was approved by the local ethics committee and has therefore been conducted in accordance with the ethical standards laid out in the 1964 Declaration of Helsinki and its later amendments.

### 2.2. Assessment Tools

Within the framework of the preparation for childbirth and during gynaecological visits at the Department of Gynaecology and Obstetrics, all patients who consented to the study were asked by a psychiatrist or intern to complete the following questionnaires:

- a survey data form: a semistructured interview to gather information on sociodemographics, the pregnancy, the family and personal psychiatric history, any psychiatric treatment carried out, the presence of stressors, and the quality of social support;
- the Edinburgh Postnatal Depression Scale (EPDS): a self-assessment questionnaire, consisting of 10 items, which revealed the presence of depressive symptoms during pregnancy and postpartum: this scale was initially designed for evaluating postpartum depression, but it can be administered even in each stage of pregnancy, as it was validated as antenatal and postpartum screening tool for minor or major depression [42]. For this study, the cut-off was 12. A score equal to or greater than this value indicates moderate to severe depression. The questionnaire was validated in the Italian version and has a high level of validity, reliability, and internal consistency [43];
- the Experience in Close Relationship (ECR): a self-administered questionnaire, composed of 36 questions, that assesses the attachment style and the way in which relationships are experienced with respect to the dimensions of Anxiety (18 items, indicating the presence of concern for relationships, in particular, the partner's availability to provide support, fear of rejection, or abandonment) and Avoidance (18 items, related to the presence of difficulties and discomfort of getting close to and depending on others): by combining the scores obtained from these two dimensions, conceptualized as a Model of the Self (Anxiety) and Model of Others (Avoidance), with reference of the underlying cognitive schemas, it is possible to distinguish four categories of attachment styles: Secure (low anxiety/low avoidance), Preoccupied (high anxiety, low avoidance), Dismissing (low anxiety/high avoidance), and Fearful-Avoidant (high anxiety/high avoidance). ECR was designed from a translation of Ainsworth et al.'s [16] descriptions of infants' attachment classifications (ambivalent, secure, and avoidant) into terms appropriate for adult love relationships. A fourth description (dismissing-avoidance, based on a similar category in the Adult Attachment Interview, the gold standard for the assessment of attachment patterns) was added later to cover the disorganized/disoriented infant-attachment category [44]. The questionnaire has a high level of validity, reliability, and internal consistency [45].

### 2.3. Sample Selection

The patients who had a EPDS score of 12 or greater were contacted by phone and invited to participate in a clinical interview at the “Perinatal Disorder Unit,” in the Department of Psychiatry of Policlinico Umberto I in Rome, with a team of psychiatrist specialized on this topic. During the clinical interview, the diagnosis was confirmed or rejected using the Structured Clinical Interview for DSM-IV Axis I Disorders (SCID-I) [46], in accordance with DSM-IV criteria. For the purpose of our study, 92 subjects were selected to enter the study group; 3 subjects were excluded for failing to give complete answers on the tests administered. Therefore, the clinical group of perinatal depression (PND) is composed of 89 subjects on the third trimester of pregnancy; these were compared to a control group (C), homogeneous by number, randomly selected (systematic sampling) from among subjects who had negative results in the EPDS (<12) and no acute psychopathological disorders.

### 2.4. Statistical Analysis

The data were analyzed using the Statistical Package for Social Science (SPSS) for Windows version 17.0. Firstly, a descriptive analysis was carried out. The data collected were examined using sociodemographic variables (age, marital status, years of schooling, and employment), pregnancy (weeks of gestation, number of pregnancies, and presence of obstetric complications), personal and family psychiatric history (previous disorders, hospital admissions, specialist visits, treatment with drugs or psychopharmacological agents, and previous peripartum disorders), patient's stress factors (conflicts with family and conflicts with partner), and anticipated support. Data are reported as frequency (%) and the age as mean ± SD (standard deviation). Clinical and control groups were compared with Student's* t*-test for quantitative variables and the chi-square test (*χ* ^2^) for qualitative ones. Pearson's correlation analysis was performed to determine the relationship between EPDS scores and Anxiety and Avoidance scores on ECR dimensions; analysis of variance (ANOVA) with post hoc test was used to evaluate differences between groups regarding the relationship between attachment styles and average scores on EPDS. A *p* value ≤ 0.05 was considered statistically significant.

## 3. Results and Discussion

### 3.1. Characteristics of the Study Population

In the PND group the average age is 32.8 years, in group C it is 33.3; 91% of PND patients are married/with a partner, as well as 93.3% of group C; there was a difference in the level of education between the two groups: 39.3% of the PND subjects and 51.7% of C subjects have a university degree, while 40.4% in the PND group and 39.3% in group C have a high-school diploma. The two groups are substantially homogeneous regarding their occupation, except for the subgroup of unemployed subjects, which was larger among the PND patients (9% versus 2.2%).

It is the first pregnancy for 59.5% of women in the clinical group and 58.4% of women in the control group. 31.5% of women with PND and 28.1% of women belonging to the control group reported the presence of complications during pregnancy; medical conditions occurring during pregnancy are present in 20.2% and 28.1%, respectively, of the PND and control group. 23.6% of PND subjects claim to be smokers, compared to 10.1% of C subjects (Table 1).

The two groups differ in a statistically significant manner due to the presence of a history of psychiatric disorders (*p* < 0.001). In the group of perinatal depression patients, 47 out of 89 women (52.8%) had a positive personal history of previous psychiatric disorders, versus 14 out of 89 women (15.7%) in the control group. In particular, in the PND group 32.9% of subjects reported having suffered from a mood disorder or depression, 40.8% from an anxiety disorder, 23.7% from an eating disorder, 1.3% from a drug addiction, and a further 1.3% from psychosis (Table 2).

In addition, eleven women in the clinical sample (12.4%) reported having suffered from psychological disorders in the perinatal period, while only two women in group C (2.2%) reported such problems. In the PND group, 31.5% of patients reported having had at least one interaction with a mental health specialist in the past, 24.7% had taken medication, 24.7% had done psychotherapy, and 4.5% of women reported a previous hospitalization in a psychiatric hospital. These percentages decrease, respectively, at 12.4%, 7.9%, 13.5%, and 0% in the control group.

**Figure 1:**
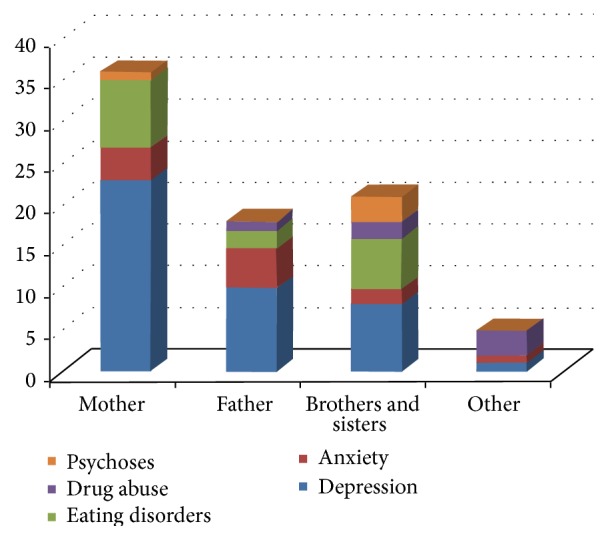
Family history of psychiatric disorders.

49.4% of patients reported a family history of psychiatric illness, compared to 30.3% of women in group C; the difference between the two groups was statistically significant (*p* = 0.009). In particular, the group of patients with a family history of psychiatric illness can attribute this, in 30.3% of cases, to patients' mothers (27 mothers); the disorders most frequently reported in the family medical history appear to be mood disorders (34.8%). Furthermore, it is worth noting that 25.8% of patients' mothers (23 mothers) had suffered from depression or mood disorders during their life (Figure 1).

In regard to the relationship with partner and family of origin, 29.2% of women with PND complained of conflicts and difficulties in relationships with their family of origin versus 7.9% of healthy women (*p* < 0.001); moreover, 32.6% of depressed women had problems with their current partner versus 4.5% of the healthy control women (*p* < 0.001).

Similarly, a difference emerges with reference to the support provided by family members in the perinatal period (65.1% of the clinical group versus 79.8% in the control group) and by partner: while 85.4% of the control group believes it can rely on the support of their partners, only 66.3% of the clinical group can say the same. Such a difference, however, does not appear significant from a statistical point of view.

### 3.2. Assessment of Romantic Attachment Styles

**Figure 2:**
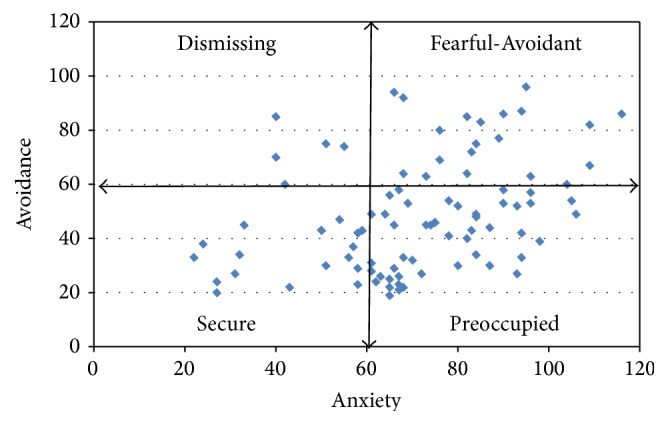
Distribution of the dimensions “Avoidance” and “Anxiety” from the ECR in PND group.

In the assessment of the romantic attachment style, revealed by the ECR, the two groups differ regarding both the Avoidance dimension (difficulty and discomfort getting close to and depending on others) and the Anxiety dimension (the presence of concern for sentimental relationships, fear of rejection, and abandonment), since the group of depressed patients has the highest average scores in both dimensions: the average on the Avoidance scale in group PND is 48.7 versus 30.4 in group C (ANOVA: *F* = 53.205, *p* < 0.001), while the average on the Anxiety scale is 71.1 in group PND versus 45.2 in group C (ANOVA: *F* = 81.015, *p* < 0.001).  Figure 2 shows the distribution of the “Avoidance” and “Anxiety” scores in the clinical group.

By combining the scores obtained from these two dimensions, it is possible to distinguish four categories or attachment styles. The two groups differ in the distribution of attachment styles evaluated by the chi-square test (*p* < 0.001), as shown in Table 3.

The most important differences are found with regards to the “secure” attachment style, which seems to be significantly higher in the control group than in the group of patients with perinatal depression (89.9% versus 41.6%) (*p* < 0.001); “Fearful-Avoidant” attachment style is, instead, better represented in the clinical group than in the control group (29.2% versus 1.1%) (*p* < 0.001).

### 3.3. Analysis of Correlation

We proceeded to evaluate the presence of a correlation between the attachment assessed by ECR and the degree of depressive perinatal pathology evaluated via EPDS, by means of Pearson's correlation analysis. The dimensions of attachment, both “Avoidance” and “Anxiety,” appear to correlate in a statistically significant manner with the severity of the perinatal depressive disorder in the group PND (“Avoidance-EPDS”:* P* Pearson = 0.338, *p* = 0.001; “Anxiety-EPDS”:* P* Pearson = 0.337, *p* = 0.001).

The romantic attachment styles relate in a statistically significant way to the score obtained from the EPDS in the PND group: there was an increase in the average score of the EPDS correlating with the increase of the level of disorganization in the romantic attachment style, as shown in Table 4. In particular, the average for patients with the “Secure” attachment style was a 15.16 on the EPDS (SD = 3.08); for a “Dismissing” attachment style it was 15.79 (SD = 4.28); patients with the “Preoccupied” attachment style had an average of 18.50 (SD = 4.14) and those with the “Fearful-Avoidant” attachment style a 19.19 (SD = 4.77) (ANOVA: *F* = 6.276, *p* = 0.001). Since the variances within the strata considered were not homogeneous, it was not possible to perform post hoc test to specify if there were significant differences between different attachment styles.

### 3.4. Discussion

To our knowledge, this study is the first to investigate the relationship between romantic attachment style, measured by ECR, and perinatal depression, evaluated by EPDS, in a cohort of pregnant Italian women.

Data collected in the current study address new information regarding the understanding of the mental state dynamics of women during pregnancy. Using ECR, we investigated the levels of Anxiety and Avoidance as well as the attachment patterns and the severity of the depressive state to verify the relevance of attachment style among the gravity of the disease.

We demonstrated that women with perinatal depression differ, in both ECR dimensions and attachment style, from the healthy control group and that these characteristics are related to the severity of the depressive state. Indeed, the group of depressed patients have higher average scores compared to the healthy control group in both Anxiety and Avoidance, respectively, conceptualized as the presence of concern for sentimental relationships, fear of rejection, and abandonment on one side and the difficulty and discomfort of getting close to and depending on others on the other side.

Regarding the romantic attachment style, the secure attachment style is significantly higher in the control group than in the group of patients with perinatal depression, while the Fearful-Avoidant attachment style is better represented in the clinical group than in the control group.

Thus, an insecure attachment model is prevalent in women affected by perinatal depression, in particular a Fearful-Avoidant pattern, followed by Dismissing and Preoccupied. Moreover, the results show that the scores on dimensions of attachment of “Avoidance” and “Anxiety” correlate with the scores obtained from the EPDS in the PND group and that there was an increase in the average score of the EPDS with the increase of the level of disorganization in the romantic attachment style.

These findings are consistent with several observations present in the literature, which suggest that insecure attachment is associated with mood disorders in general [29]. However, we have been able to extend them to a specific life situation (pregnancy) in a cohort of Italian women, corresponding with recent international studies that investigated the role of maternal attachment in the transition to parenthood [31–36].

Furthermore, this study is the first to demonstrate that the severity of depression (measured with the EPDS) increases in proportion to attachment disorganization, reaching the highest score in patients with a Fearful-Avoidant attachment, followed by those who have a preoccupied attachment and finally a dismissing one.

This means that perinatal depression will be experienced more severely as more negative the Model of Self (Anxiety on ECR) and the Model of Other (Avoidance on ECR) are. These models also influence the quality of close relationships, with family of origin and with their partner. In our study, both “conflict with the partner” and “conflict with the family of origin” appear to differ in a statistically significant manner between the two groups, as well as expectations that depressed women have of receiving support from their partners and their families of origin.

According to Bowlby's theory [17–20], the avoidant subject, whose attachment pattern has been developed based on distancing strategies, withdrawal, and deactivation and who has therefore learned, at least in appearance, to do without a reference point, will experience a greater conflict with the family of origin or with their partner, as they are unable to rely and depend on others. Faced with conflict, the avoidant subject will tend to confirm a feeling of self-sufficiency by evading the relationship itself; on the other hand, the anxious-ambivalent subject, fearful of rejection and abandonment, will tend to put in place a number of demands in order to obtain confirmation and reassurance, dominated by a constant concern for relationships, in particular regarding the availability of the partner to provide support [47].

Therefore, subjects with insecure attachments have difficulty in establishing a torque relationship that becomes a relationship based on reciprocity, in which each of the two components is able to be “subject” and “object” in a relationship of mutual dependence and exchange. The partnership can therefore represent both the place where one confirms the Internal Working Models that have already been tested in the past or an opportunity to confront and question unresolved aspects of the past in an adaptive renegotiation, which allows for greater separation and differentiation of the self. If this separation and identification of the self fail to be reached, it will be easier to slip into a depressive condition.

Even if our goal in this study was to point out a particular kind of psychological risk factors—the quality of attachment—from all the data obtained in the present work, it is possible to outline a framework of biopsychosocial risk factors that are consistent with the literature [8, 48, 49]. Above all, the most recent studies underline a past history of depression, anxiety, or bipolar disorders, as well as psychosocial factors, such as ongoing conflict with partner, poor social support, and ongoing stressful life events, as risk factors [50–53].

Our results confirm the presence of psychiatric disorders (especially regarding the domain of anxious-depressive disorders) in the personal medical histories of the new-mothers, who develop perinatal depression, such as a family history of psychiatric disorders. In particular, it is interesting to note the recurring presence of depression in patient's mothers, in a kind of trans-generational transmission of the affective disorder, which has its roots in both biological and relational substratum. Indeed, the parental role is an event in the life of a woman that closely relates to the domains of attachment, in which there is a reactivation of representations of attachment experienced with their own parents and, therefore, a considerable permeability to the emergence of experiences related to caregiving and to their own relational history. Based on how those experiences were lived and elaborated, the woman will shape her “feeling of motherhood.” Therefore, the experience with motherhood that every woman had (including the mother's depression) could affect the level of adaptation and reorganization of the relationship that occurs at both intrapsychic and relational level during pregnancy [54–56].

## 4. Conclusion

This study reports the first phase of a longitudinal study designed to identify potential predictors (such as attachment style and psychopathological vulnerability) to the development of perinatal depression. In order to propose more effective strategies for identification and early intervention in perinatal depression, we recommend recognising the attachment pattern as an important factor in the development of depressive symptoms during transition to motherhood. By addressing the mother's unresolved attachment conflicts with an attachment based psychotherapeutic intervention, it is believed that the development of a more adaptive parenting and a more secure and less disorganised attachment between the mother and her infant is facilitated, as well as a better couple relationship [57].

This study had several limitations. First, we evaluated women's mental states with self-administered questionnaires, even if they have satisfactory psychometric qualities. In particular, ECR was chosen instead of Adult Attachment Interview (AAI), the gold standard for the evaluation of attachment patterns, to increase sample size, considering the difficulties associated with the administration of the AAI. In principle, the two measures might have been substantially associated, but in fact they seem to be only moderately related: few studies have found the AAI to be related to marital relationship quality and a few have found self-report romantic attachment measures to be related to parenting; therefore, authors conclude that attachment measures are more precise when analyzed in terms of dimensions rather than types [58, 59]. Secondly, histories regarding mood disorders before pregnancy and family psychiatric history were not assessed using diagnostic tools. Finally, the significant relationship between perinatal depression and Fearful-Avoidant attachment style found in this study is cross-sectional.

In the future, it is mandatory that we deepen our understanding of these preliminary findings with an increased sample size and a long-term prospective follow-up, to study the differences in attachment patterns between depressed and healthy women after delivery (second phase of study). Furthermore, it could be useful to include, in the assessment tools, the Adult Attachment Interview and to compare it with ECR results. Finally, it could be interesting to plan further studies that take into account the characteristics of the partner and the role that it can play as a protective factor or as a risk in the development of perinatal depression.

Such observations will allow us to identify potential predictors for the development of psychopathology in perinatal period and to plan preventive interventions focus on attachment and relational patterns during prenatal psychoeducational courses for the “at risk” population.

## Figures and Tables

**Table 1:** Descriptive analysis of both groups: sociodemographics variables related to pregnancy (*t*-test).

Variables	PND	C	*p* value
	(*N* = 89)	(*N* = 89)	
Mean age (SD)	32.8 (5.7)	33.3 (4.8)	NS
Civil status (%)			
Single	4.5	3.4	NS
Married∖common-law wife	91	93.3	NS
Separated∖divorcee	3.4	3.4	NS
Widow	1.1	0	NS
Socioenvironmental position (%)			
Single	3.4	0	NS
Family of origin	9	2.2	NS
Her own family	86.5	95.5	NS
Other	1.1	2.2	NS
Schooling (%)			
Junior high-school diploma	20.2	9	NS
Senior high-school diploma	40.4	39.3	NS
University degree	39.3	51.7	NS
Working position (%)			
Student	3.4	4.5	NS
Housewife	12.4	12.4	NS
Unemployed	9	2.2	NS
Entrepreneur∖freelancer	11.2	14.6	NS
Employee∖executive	46.1	49.4	NS
Worker and the like	14.6	14.6	NS
Casual work	3.4	2.2	NS
First pregnancy (%)	59.5	58.4	NS
Complication during pregnancy (%)	31.5	28.1	NS

PND: perinatal depression group; C: control group. *N*: number of subjects. NS: not significant.

**Table 2:** Comparison of personal psychiatric history of both groups (*χ* ^2^).

Psychiatric history (%)	PND	C	*p* value
	(*N* = 89)	(*N* = 89)	
Presence of psychiatric disorders	52.8	15.7	<0.001
Mood disorder	32.9	7.9	NS
Anxiety disorder	40.8	5.6	NS
Eating disorder	23.7	3.4	NS
Drug addiction	1.3	0	NS
Psychosis	1.3	0	NS
Previous perinatal disorder	12.4	2.2	NS

PND: perinatal depression group; C: control group. *N*: number of subjects. NS: not significant.

**Table 3:** Comparison of romantic attachment styles in the two groups (*χ* ^2^).

Romantic attachment styles (%)	PND (*N* = 89)	C (*N* = 89)	*p* value
Secure	41.6	89.9	<0.001
Dismissing	15.7	3.4	NS
Preoccupied	13.5	5.6	NS
Fearful-Avoidant	29.2	1.1	<0.001

PND: perinatal depression group; C: control group. *N*: number of subjects. NS: not significant.

**Table 4:** Correlations between romantic attachment styles and the EPDS score in both groups (*∗*).

Romantic attachment styles	PND (*N* = 89)		C (*N* = 89)	
	*N*	EPDS mean score (SD)	*N*	EPDS mean score (SD)
Secure	37	15.16 (3.08)	80	5.53 (3.18)
Dismissing	14	15.79 (4.28)	3	7.33 (2.08)
Preoccupied	12	18.50 (4.14)	5	7.40 (2.61)
Fearful-Avoidant	26	19.19 (4.77)	1	3 (0)

PND: perinatal depression group; C: control group. *N*: number of subjects.

^*∗*^ *p* value = 0.001; ANOVA: *F* = 6.276; SD = standard deviation.
